# Accuracy Evaluation of Dense Matching Techniques for Casting Part Dimensional Verification

**DOI:** 10.3390/s18093074

**Published:** 2018-09-13

**Authors:** Gorka Kortaberria, Unai Mutilba, Eneko Gomez-Acedo, Alberto Tellaeche, Rikardo Minguez

**Affiliations:** 1 Department of Mechanical Engineering, IK4-Tekniker, 20600 Eibar, Spain; unai.mutilba@tekniker.es (U.M.); eneko.gomez-acedo@tekniker.es (E.G.-A.); 2 Department of Smart and Autonomous Systems, IK4-Tekniker, 20600 Eibar, Spain; alberto.tellaeche@tekniker.es; 3 Department of Graphic Design and Engineering Projects, University of the Basque Country, 48013 Bilbao, Spain; rikardo.minguez@ehu.eus

**Keywords:** point cloud, uncertainty, dense matching, photogrammetry, inspection, multi-view stereo, structure from motion

## Abstract

Product optimization for casting and post-casting manufacturing processes is becoming compulsory to compete in the current global manufacturing scenario. Casting design, simulation and verification tools are becoming crucial for eliminating oversized dimensions without affecting the casting component functionality. Thus, material and production costs decrease to maintain the foundry process profitable on the large-scale component supplier market. New measurement methods, such as dense matching techniques, rely on surface texture of casting parts to enable the 3D dense reconstruction of surface points without the need of an active light source as usually applied with 3D scanning optical sensors. This paper presents the accuracy evaluation of dense matching based approaches for casting part verification. It compares the accuracy obtained by dense matching technique with already certified and validated optical measuring methods. This uncertainty evaluation exercise considers both artificial targets and key natural points to quantify the possibilities and scope of each approximation. Obtained results, for both lab and workshop conditions, show that this image data processing procedure is fit for purpose to fulfill the required measurement tolerances for casting part manufacturing processes.

## 1. Introduction

The present study was focused on the quality assurance requirements and measurement procedures of the foundry-based manufacturing processes and can also be applied to other similar manufacturing processes such as the forge, where manufacturing tolerances may be similar. Nowadays, large scale raw component suppliers are requesting the optimization of their productive processes to compete with lower cost manufacturers worldwide. This optimization demands more efficient and profitable manufacturing methods to control the stability of the processes through procedures that guarantee the quality of the products. Following this objective, a key stage in the manufacture value chain of wind components is the casting phase. Here, the pre-form of the parts that are subsequently machined is generated to obtain their definitive functionality at the operational level. Therefore, casting parts should ensure a minimum oversize that ensures that material exists in those areas of the piece to be machined. Simulation tools such as Computer Aided Design (CAD) software and Finite Element Analysis (FEA) software are employed targeting this aim. Nevertheless, realistic models of the casting process are hardly established due to the complexity of the manufacturing process and the understanding of the involved parameters. Thus, part inspection becomes crucial to verify that the manufacturing process accuracy is under manufacturing tolerances and these results are also used to adjust simulation models as well as to compensate the pattern’s design and size. In this scenario, casting quality characterization encompasses five main categories: casting finishing, dimensional accuracy, mechanical properties, chemical composition and casting soundness. All aspects are crucial to assure that the part meets customer’s specifications and non-destructive testing methods (visual inspection, measuring machines, magnetic particle inspection, ultrasonic testing, etc.) are usually applied for their characterization.

An accurate dimensional inspection is commonly applied for First Article Inspection (FAI) or even for serial production control, where commonly part-to-CAD analysis and/or geometrical analysis according to ISO 8062-3 Standard is required for manufactured parts conformity assessment.

Either the casting manufacturing processes or the measurement procedures and technologies have evolved to speed up the result obtaining process to feed faster the production quality control stage. Thus, currently, a clear tendency aims to integrate in-situ measurements during the manufacturing process to substitute final closeout inspections. However, total time consumption for part inspection should be tackled to offer a rapid response. Here, two stages can be clearly distinguished: the acquisition and the processing of the measures.

Nowadays, technologies applied in the verification process do not allow a total automation of the inspection stage for several main reasons: the dimensions of the pieces, the multiple stations (positions) of the inspection systems as well as their lack of autonomy. Therefore, there is a latent opportunity in terms of improving existing procedures to reduce inspection times. In this scenario, the present paper highlights dense matching techniques to solve the aforementioned problem.

### 1.1. Background

**Figure 1:**
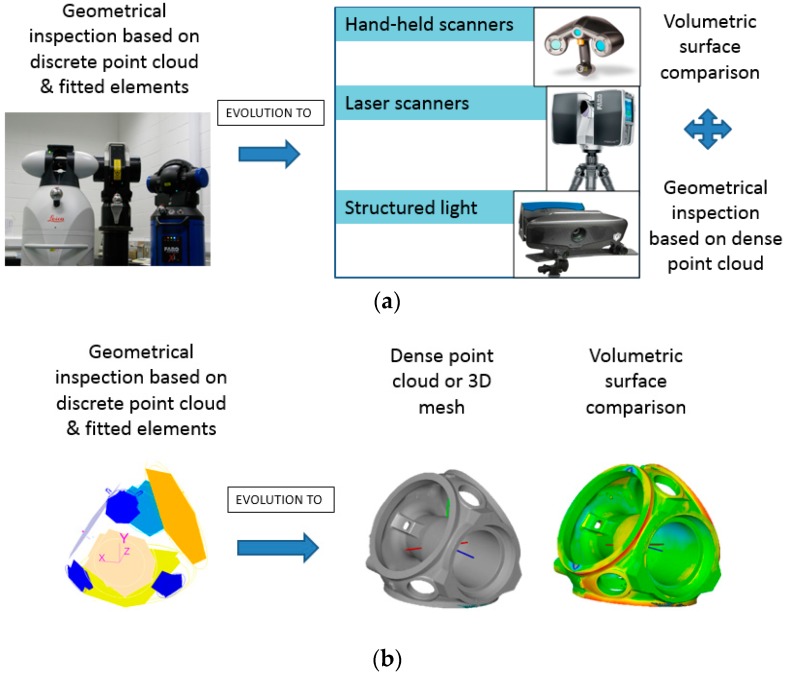
Evolution of measuring technology approaches for casting part dimensional verification: (**a**) data acquisition technologies; and (**b**) data processing schemes.

The state of the art of 3D measuring technologies and methodologies for casting part verification have substantially been developed in the last few years, offering faster, more complete and more automatic approaches. The evolution of measurement methodologies is closely related to the employed measurement technologies and the output data that they provide. To understand the portfolio of common measuring technologies for medium to large scales components [1,2,3] an overview is presented in Figure 1, where an evolution from contact to non-contact approaches is presented and also a data processing procedures evolution is shown. The difference and the use of each measuring technology depends on the measurement case study and the dimensional analysis required in each case. Therefore, the selection of the most appropriate technology depends on the measurable requirements such as: measuring uncertainty, point resolution, type of processing, measuring scale, part accessibility, etc.

Traditionally, casting part dimensional verification has been executed by handheld manual instruments such as calipers, calibrated fixtures or measuring tapes. However, these devices do not allow verifying the main dimensions of large parts which leads to partial verification with low process quality confidence. To overcome this limitation, large Coordinate Measuring Machines (CMM) and Portable Coordinate Measuring Machines (PCMM) were developed many years ago pushed by large part manufacturers. CMMs are the most accurate approach but they require bringing the part to the machine which is time-consuming and sometimes an unaffordable option due to part dimensions. PCMMs aim to cover this physical limitation. Nowadays, many kinds of systems and brands exist [4,5]. A review of 3D measuring systems is also presented related to manufacturing verification needs in several scales and suitability of measuring devices and techniques is studied for 3D parts.

**Figure 2:**
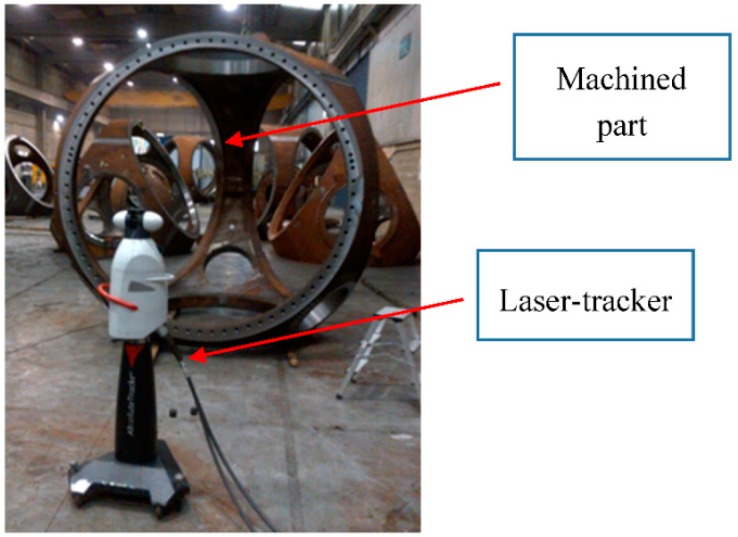
Laser-tracker technology for machined part dimensional inspection.

Laser tracker technology was the pioneer in volumetric eolic component verification (Figure 2). Its parametric modeling is mentioned in [6,7], whereas a general review of its performance and applicability is presented in [8]. It has been widely used for machined component inspection for almost 20 years, thus its application to casting part inspection is imposed by machining companies in the eolic component manufacturing chain. It offers the possibility to quantify and verify the overall dimensions of the part with high accuracy requirements as well as allows verifying the accuracy performance of other measuring or manufacturing means [9,10,11,12]. Recent laser-tracker developments include hand-held accessories to scan the part surface without contact by means of real-time tracking of a laser-triangulation sensor or probe and even highly automated scanning robotic approaches. Compared to initial laser tracker technology that only offers the possibility to measure a discrete point cloud touching the part surface with a calibrated retroreflector, automatic eolic component inspection procedures come closer to the goal of surface scanning.

**Figure 3:**
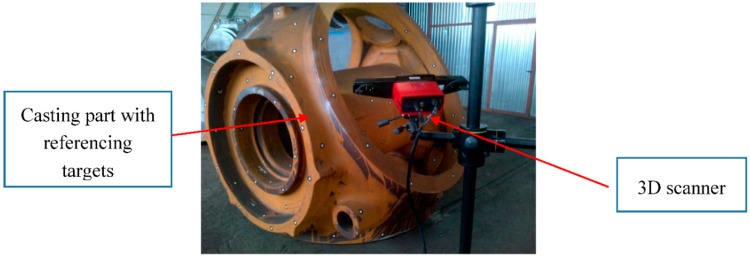
Scanning of casting part by means of structure light scanner and reference targets.

Conventional shape and dimensional analysis of casting components with tactile coordinate measuring systems has some limitations, such as: discrete point measurement strategy, lack of free-form shape information and time consumption for volumetric component measurement. In the last few years, 3D optical scanners [5] are replacing laser trackers as the most used measuring equipment for the dimensional quality control assessment of machined and casting parts. Modeling and calibration issues are mentioned in [13,14,15,16], whereas some examples of application are studied in [17,18] and even accuracy performance is determined for lighting conditions in [19]. Although their accuracy is still a bit lower (±30 µm/m) than laser-tracker technologies (±15 + 6 µm/m) for high accuracy applications, casting part verification methodologies have been adapted to these technologies as they offer a volumetric view of the part compared to its nominal CAD definition. Optical scanners such as laser-triangulation sensors [20], structured light sensors (Figure 3), or even Time Of Flight (TOF) based laser- scanners [21,22] have the ability to measure dense point clouds that are used to analyze the oversized material distribution or carry out reverse engineering tasks. They capture more detailed and easily interpretable quality information of an object with significantly shorter measuring times and accuracies ranging from 0.05 mm to 2–3 mm for large parts (several meters) which assures post-machining steps. Many technology suppliers already offer this kind of equipment as an alternative to tactile approaches.

The main drawbacks of these optical systems are data acquisition time and part accessibility. As the part is usually bigger than the field of view (FOV) of the measuring system and the required point cloud resolution is quite demanding, the scanning system needs to be located in several positions around the part to scan every surface point. This means that each device location should be accurately aligned to previous locations with common references to reconstruct the 3D point cloud in a common coordinate system. However, these partial alignments add inaccuracy to overall point clouds as a residual error is carried out each time that can suppose a non-continuous point cloud, especially for round parts. Besides, this working procedure forces to see common reference targets among different consecutive scanner locations, which makes the measuring procedure a time consuming and restricted method. Currently, to tackle and enhance the accuracy of device location alignment stage, structured light based scanners are combined with photogrammetric approaches which guarantee higher accuracy referencing points. This method comprises two measuring systems and therefore steps. First, a photogrammetric approach is used to measure some dot targets all around the part and these artificial targets are used as a reference for the subsequent scanning step. The points are identified and used as tie points for each partial scanning, enabling to fuse all 3D data in a common reference system. Moreover, the accuracy is improved as the output of the previous photogrammetric step is more precise than locating the scanners in multiple locations and applying consecutive point cloud fusions which leads to overall 3D point cloud inaccuracies. Besides, using previously calibrated dot targets as a reference enables taking partial scans all around the part without having to assure a consecutive overlapping among each scan and consequently offering a more flexible measuring procedure. Another possibility to improve data acquisition time is to use robotic measuring cells. Nevertheless, this approach is nowadays oriented to other manufacturing sectors where required investment can ensure a fast-economic return.

X-ray computed tomography (CT) is a new technology that offers not only the output of 3D point cloud of the surface but also the inner 3D structure for material integrity checking. However, this technology is currently limited to 1 m range due to required installation costs and X-ray source power. Some references regarding to technique description, modeling, accuracy and applicability are mentioned in [22,23,24,25].

**Figure 4:**
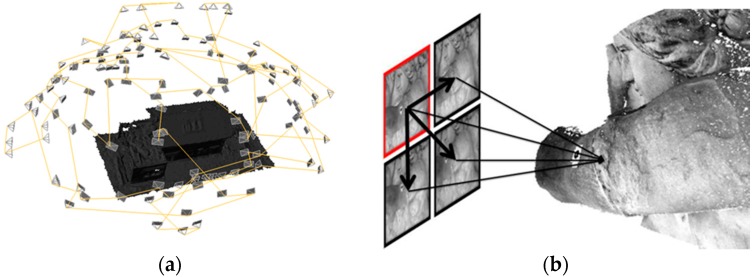
Dense reconstruction workflow among multiple views: (**a**) photogrammetric step; and (**b**) dense matching approach.

Dense matching techniques are the development of vision-based systems to measure dense 3D point cloud from imagery data. It comprises two main stages (see Figure 4) to reconstruct the dense point cloud: camera network calibration and dense image matching based on previously estimated photogrammetric results. In the literature, network orientation is also referred to as a structure from motion technique [26,27,28,29]. It targets going beyond traditional photogrammetric solutions improving image processing algorithms for textured surface reconstruction. Therefore, it requires texturized surfaces which means that non-homogeneous images are needed to feed and solve feature-based matching problem [30], stereo pair triangulation and multi-view bundle block adjustment [31,32,33,34].

On the one hand, the photogrammetric stage allows sorting out the bundle adjustment of the photogrammetric model the results of which are: 3D point coordinates (XYZ) by means of multi-view triangulation, self-camera calibration (intrinsic parameters) and orientation and position of the images (extrinsic calibration) according to the main coordinate system. These output values are afterwards the input data for dense matching approaches [35]. The photogrammetric stage can be carried out with coded targets [36] as well as based on natural tie points [37].

On the other hand, dense matching techniques [38,39,40] accomplish 3D dense point cloud reconstruction from oriented images and considering known camera intrinsic parameters. Tie points are combined in a bundle block adjustment, resulting in the 3D coordinates of all tie points and, more importantly, the position and orientation of each image. If these parameters are previously and accurately estimated, the expected results for dense matching approaches are supposed to be more precise. An accuracy study of this reconstruction method and algorithms is presented in [41,42]. Matching techniques [43,44] are usually applied in two steps (see Figure 4) to obtain at first sparse matching points (few key points) and then to use them as input for dense matching and triangulation (dense point cloud reconstruction). Matching algorithms are based on known relative orientations among images and image-pair searching schemes. For each image pixel on a reference image, a corresponding point is established in other images and views by means of pixel-based matching algorithm [38,39,40]. Once the dense correspondence is solved, stereo or multi-view triangulation [45] is enabled and determined, which offers 3D colorized dense point clouds from epipolar images and constraints [41,42,43,44]. Most e approaches are based on the minimization of cost functions that consider the degree of the similarity among pixels and includes constraints to consider possible errors in the matching process as well as geometric discontinuity changes.

An increasing number of software solutions for the automatic or semi-automatic generation of textured dense 3D point clouds images have recently appeared in the market [46,47,48]. iWitnessPRO-Agilis© photogrammetric software has evolved to dense reconstruction capabilities. It combines a photogrammetric library (Australis©) and the SURE© tool from the Institute For Photogrammetry at the University of Stuttgart to enable 3D point cloud reconstruction with different camera network calibration approaches (semi-automatic, automatic with coded target, and targetless orientation by means of feature-based matching) [49,50].

### 1.2. Motivation

Although dense matching photogrammetric based applications are extending more and more into industrial applications, the casting part verification with these novel photogrammetric methods has not been studied before. Most applications target cultural heritage, aerial topography or satellite surveying/mapping applications, but not industrial applications. Thus far, these approaches have not been studied from the point of view of accuracy and traceability, because there is not much information in the current literature [51,52]. It is within this context that this paper aims to provide an approach to assess the accuracy of dense matching techniques from a metrological point of view. Therefore, the approach adopted here is based on established and certified measuring approaches whose accuracy is already known and is used as a reference.

Considering that usually employed certified methods combine photogrammetry with further scanning processes aiming to increase point cloud accuracy and surface continuity, this study established the scope of dense matching techniques as an alternative verification approach employing a unique hand-held industrial camera. If the imagery data from photogrammetric approaches are also suitable to obtain dense point clouds, there is no need to scan the part after the photogrammetric stage has been carried out, which would considerably shorten the overall measuring time and reduce the cost of the measuring solution and technology.

The reduction of the measuring process duration would enable lower cost measuring services and faster feedback for casting part manufacturers to improve the process quality control for high value parts.

### 1.3. Objectives

The objectives of this research were to understand the accuracy assessment and measuring process requirements for dense matching photogrammetric techniques focused on the verification of medium to large casting parts. One remarkable aspect, in addition to sufficient accuracy to fulfill the manufacturing tolerances, is the time consumption required to carry out the dimensional verification of the parts. To consider this novel method as a suitable procedure, the overall process should reduce time consumption at least during the image data acquisition stage. This point led to carrying out a comparison of automated reconstruction approaches based on coded-targets or target-free schemas. Other factors such as adaptability of the process for different part dimensions and part complexity were also considered in this research.

Targeting the above-mentioned goals, a certified method for 3D verification was considered as a reference to quantify, determine and compare the achieved results between both measuring procedures and therefore to validate the studied approach.

Apart from these objectives, this study also focusede on the adaptability and improvement of dense matching techniques for the case presented, assessing the pros and cons of this method against current state of art approaches.

## 2. Materials and Methods

### 2.1. Measurement Method

Dense photogrammetry is an image processing technique based on 2D images to obtain 3D information based on the texturing of the surface to be measured. The multiple images that ensure high overlapping areas are processed with a photogrammetric analysis software to obtain a dense point cloud. The software employed in this research is called iWitnessPRO-Agilis©. This software is a photogrammetric tool (iWitnessPro©) enhanced with dense matching capabilities (SURE© tool) to construct dense point cloud estimation based on image data.

To execute dense photogrammetry, it is necessary to have a part with well-conditioned surface texture such as casting or forged parts. Without this texture, the dense reconstruction of the surface by means of image processing is not possible. Homogeneously texturized parts, such as machined components, cannot be measured with this method.

**Figure 5:**
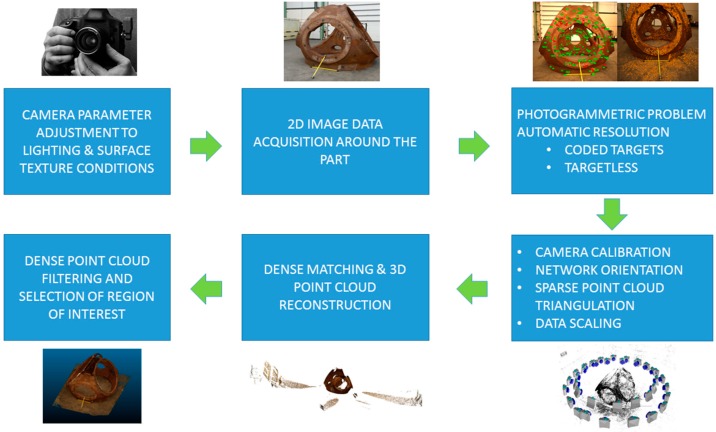
Dense matching process detailed workflow for shop-floor case study.

This research aimed to allow as automatic as possible data processing, thus, in addition to the textured surface, coded targets were placed on the measurand to facilitate this aim. The approach considering only natural key points (feature-based matching-approach) is offered by the software but the processing requires users few controls such as data scaling. The location of coded targets are recommendable to solve with precision the following aspects: the calibration of the camera itself (intrinsic parameters), the external orientation (extrinsic parameter) of each image obtained and the spatial XYZ coordinates of each target whose center is to be determined. The technique also allows sorting the photogrammetric problem out without coded targets if the part offers characteristic geometric elements that can be discerned in several images as reference unequivocal points. However, it is expected that the accuracy is reduced if the use of targets is avoided, thus advantages and disadvantages of each approach should be considered. To automate the processing of the images, it is necessary for the software to independently calculate the external orientation, internal and scaling of the images, in addition to executing the dense reconstruction from these known data. To guarantee this result, the software performs two main stages. First, the photogrammetry is solved and then the dense matching technique is applied to obtain dense point clouds. This dense matching technique employs photogrammetric results to establish the correspondence among images based on image neighborhood data (average pixel values) and define the surface points by means of a combination of multi-stereo pairs and their triangulation for all the images. The overall workflow that comprises both processing stages is shown in Figure 5.

To guarantee accurate results, both photogrammetric and photography requirements have to be considered. If only photogrammetric camera set-ups are used, the surface points are not well focused, and the dense matching is not working. Therefore, a balance between both fields should be considered when image acquisition is carried out. A more detailed definition of the measuring process and the data processing follows these steps and scheme:

1. Preparation of the cast part with targets (if necessary) and a calibrated length bar
2. Adjustment of the camera and lens to measure the measuring scenario
3. Image acquisition process with fixed camera settings
4. Data processing
  (a) Photogrammetry with coded targets or targetless approaches
  (b) Dense matching
5. Dense point cloud filtering and refinement

In each step, multiple aspects should be analyzed before the measurement to assure that the overall measurement workflow will be successful. The most critical ones are listed below:

- Camera and lenses adjustment: It defines the resolution, the depth of field, exposition time, lenses aperture, the contrast of surface tie points, etc.
- Image network design: Minimum overlapping among images both for photogrammetry and dense matching reconstruction is necessary to detect coded targets, key points or unique surface points.
- Camera self-calibration: Intrinsic parameters (focal length, principal point, optical distortions, etc.) are determined for each measurement as camera internal parameters are not temporally stable.

Thus, the edge-cutting method presented in this research requires a good combination of these influencing factors to obtain suitable results. In the following paragraphs, a detailed description of employed adjustments are defined for the developed measuring method.

#### 2.1.1. Preparation of the Scene

The criteria for the placement of the coded targets (based on dot distribution) are the following:

- Minimum five common targets between different images
- Robustness is improved if there are eight or more targets
- Size of the target should ensure an approximate image size of 10 pixels
- Image processing module of the software cannot detect another type of coding, which is the reason to use this type of targets. They can be white on black or red on black.

**Figure 6:**
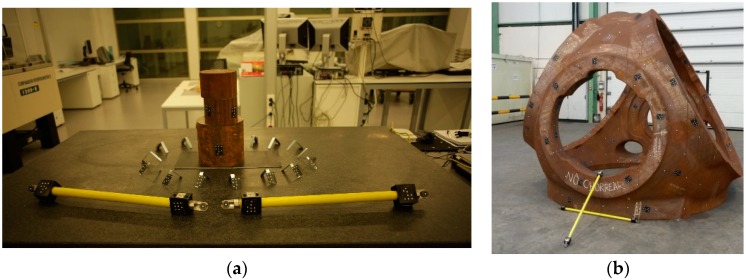
Coded targets and calibrated scale bar located around the textured part: (**a**) lab testing; and (**b**) shop-floor testing.

In addition to the targets, it is necessary to locate on the scene to be measured a calibrated length bar (see Figure 6) that establishes the scale of the acquired images. If there is more than one artifact, the scaling is performed with the average value adjusted for both measurements. It is interesting for the scaling to be automatic to use a bar with encoded targets so that the software in question (iWitnessPRO-Agilis©) knows the points to which the scaling corresponds. It is necessary to make this definition in the software at least once.

#### 2.1.2. Camera Adjustment

Once the scene is prepared, it is necessary to adjust the camera parameters to the lighting conditions of the room where the images are taken. Normally, in a photogrammetric process, the camera adjustment is set on the targets to be measured (maximum aperture and a zoom of 10×), but in a dense matching it is also necessary to consider a good contrast on surface points to be scanned. Therefore, when adjusting the camera, a compromise should be found between the coded targets and the surface texture to be measured. The main parameters to be adjusted are:

- Working distance
- The aperture
- Flash intensity (if required)
- Depth of field

These parameters should be kept constant for the entire measurement to solve the photogrammetric bundle adjustment.

The identification of the camera with the software is automatic and therefore the parameters related to the sensor (resolution and image dimension) are determined automatically. However, it is necessary to verify these parameters and change them if necessary. In this study, a Canon EOS-1Ds Mark III camera was used with 25 mm focal length lenses.

#### 2.1.3. Acquisition of Images

When it comes to image acquisition process, it is necessary to consider what is intended to be measured both with photogrammetry and dense matching techniques. Therefore, in addition to photogrammetric aspects, a proper contrast and triangulation of surface points should be guaranteed. For this, it is necessary to reach a compromise between a good angle of triangulation and an excessive change of perspective between consecutive images. Thus, high angular views should be avoided and angles ranging from 60° to 110° are recommended. Depending on the relative angle between pairs of images, the accuracy of the photogrammetric or dense reconstruction stage will be reinforced.

In addition to triangulation, it is important to take care of the internal calibration aspect, for which it is necessary to acquire rotated images on the image plane. Normally, 4 × 90° rotated images are acquired, respectively, at the beginning of the measurement. The same criteria of acquiring rotated images during the measurement is strongly recommended to enhance camera self-calibration.

**Figure 7:**
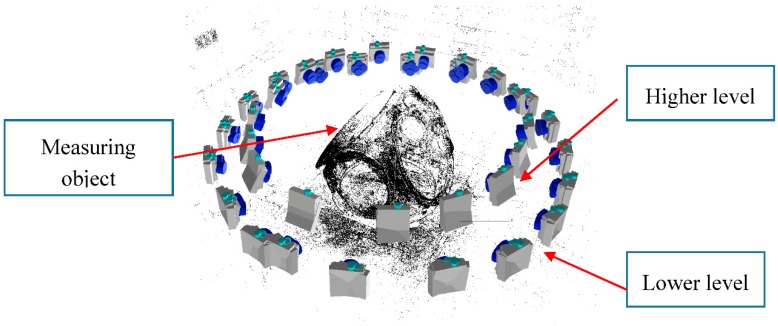
Camera shooting technique around the part.

In this study, the images were taken around the part following the clockwise criteria and combining different height and view angles for each image acquisition position (see Figure 7).

#### 2.1.4. Image Data Processing

Once the images are acquired, the processing of the photogrammetry and dense matching is carried out in two steps. The bundle adjustment is automatically solved if coded targets are employed for determination of image orientation and scaling, while the processing is more manual when natural key points are determined from imagery. The second step, named dense matching, is supposed to be automatic, but depending on the image data casuistic, few parameters should be fitted, such as: density of point cloud, image-pair combinations, pixel point neighborhood size, etc.

The extension of the generated file format for dense point cloud is “las” extension, therefore CloudCompare© software is used to visualize the point cloud and to convert it to more traditional “ascii” format files as well as to select the region of interest (ROI) of the dense point cloud. These files are afterwards used for the comparison of measured against reference data.

#### Photogrammetric Processing

• *Based on coded targets*

This is the most common method to solve the bundle adjustment in industrial photogrammetric systems as the automation level is high. Only preliminary image processing parameters need to be adjusted to assure solving intrinsic orientation, extrinsic orientation and coordinates of 3D point of interest at the same time without knowing a priori values of these parameters.

**Figure 8:**
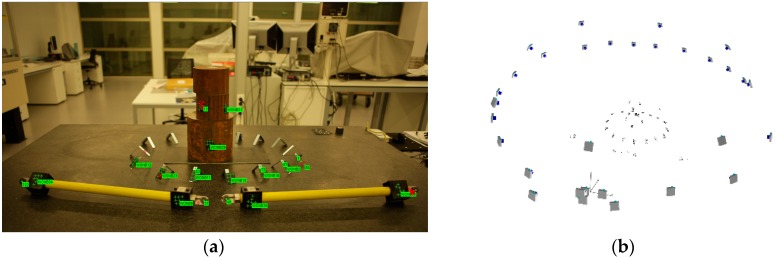
Automated orientation in lab: (**a**) coded target image identification (in green); and (**b**) sparse triangulation of coded targets and camera network determination.

The coded targets enable the automatic referencing between image pairs and among every image rejecting outliers and obtaining more robust image correspondence as the point identification is unequivocal. Moreover, the 3D scene scaling is carried out, automatically identifying the coded targets that correspond to the calibrated length bar (see Figure 8). The main drawback of this approach is the preparation of the scene with the artificial targets and the assuring of a minimum number of common points among images.

• *Based on Feature Based Matching (FBM)*

It is a different photogrammetric processing approach to estimate the extrinsic calibration of images. Instead of using artificial coded targets, natural key features are identified in the images and the correspondence among the multiple stereo pairs is estimated by means of image matching techniques. Approximately 50,000 feature points are used in each image. After establishing the tie points among the images, the relative orientation between image pairs is determined and the absolute orientation considering the first image as a reference. Finally, a bundle adjustment (minimum convergence of three image rays) approach is applied to refine and improve the extrinsic calibration of the camera network.

**Figure 9:**
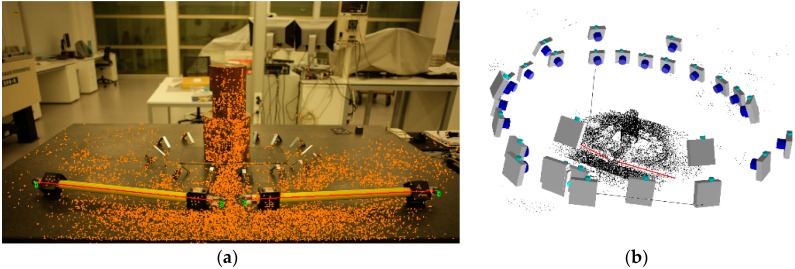
Targetless orientation in lab: (**a**) tie point image identification (in orange) and length bar definition (in red); and (**b**) sparse triangulation of feature points and camera network determination.

Once the camera network is defined, 3D scene scaling is established, which is necessary to obtain a metric 3D point cloud. This step is manually executed by choosing an image pair where the calibrated length bar is seen. Based on manual target centroiding identification and epipolar image correspondence, the image points corresponding to the artifact were selected and applied to a known calibrated distance (see Figure 9).

#### Dense Matching Reconstruction

**Figure 10:**
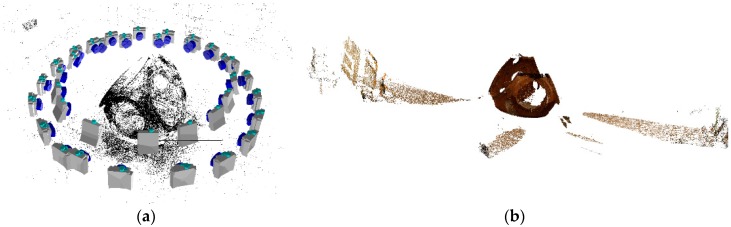
Dense matching schema for shop-floor case study: (**a**) structure from motion; and (**b**) dense 3D colorized point cloud without filtering.

The dense matching reconstruction is performed by means of SURE© software, which is a tool for photogrammetric surface reconstruction from imagery. It is a software solution for multi-view stereo, which enables the derivation of dense point clouds from a given set of images and known orientations (see Figure 10). Nearly one 3D point can be obtained corresponding to each image pixel with the highest resolution.

The input to SURE is a set of images and the corresponding interior and exterior orientations (structure from motion approach), whereas the output information of the software is dense point clouds or depth images. It is a multi-stereo solution, so single stereo models (using two images) are processed and subsequently fused. It automatically analyzes the block configuration to find suitable stereo models to match in several steps (coarse methods for stereo pair selection and accurate methods for dense stereo 3D reconstruction). During the 3D reconstruction step, the disparity information from multiple images is fused and 3D points or depth images are computed. By exploiting the redundancy of multiple stereo pairs and combinations, blunder and outliers are filtered or rejected. In this research, the full resolution of the camera (21 Megapixels) was used and a point was triangulated as minimum with a stereo pair.

### 2.2. Validation Procedure for Accuracy Assessment

The validation of the presented methodology was performed by means of a “two-step” measuring procedure combining an optical 3D scanner (ATOS IIe©, Braunschweig, Germany) based on structured light with an optical portable CMM (TRITOP©, Braunschweig, Germany) based on photogrammetry, which assures high accuracy reference data used for the comparisons presented in this research. The fusion of these technologies enables obtaining higher accuracy point cloud data (grown truth) for medium to large dimension parts and a more flexible scanning procedure.

**Figure 11:**
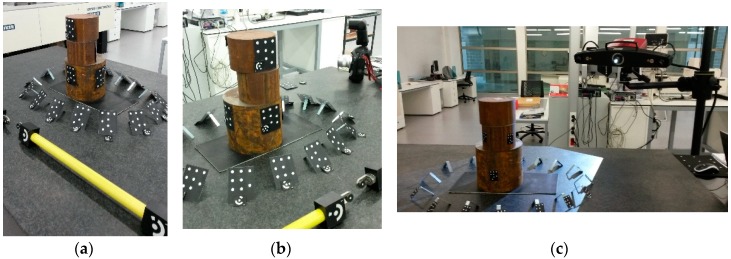
Measuring method for reference data determination in lab: (**a**) scene preparation with coded targets (ring and dot type) and scale bar; (**b**) photogrammetric camera (TRITOP©); and (**c**) structured light 3D scanner (ATOS IIe©).

First, a photogrammetric procedure was applied to measure the XYZ coordinates of all the targets placed in the measuring scenario (see Figure 11a). This was accomplished by TRITOP system employing the ring coded targets, a high-tech industrial camera (see Figure 11b), and advanced photogrammetric software to solve the bundle adjustment problem. Dot type coded targets were used as fiducial references for the subsequent scanning stage as the TRITOP system considers these targets as non-coded targets. Each partial scan was referenced by means of these fiducials to a common reference system defined by previously executed photogrammetric stage. The targets based on dot type codification were also used for the automated orientation case study in further analysis presented in this paper. Therefore, both types of coded targets (see Figure 11a) were placed in similar locations to guarantee that the photogrammetric problem can be solved and the reachable accuracy is practically the same.

Once that photogrammetry was solved, a dense 3D scanning measurement of the part was executed with the available structured light 3D scanner (see Figure 11c) using the dot type fiducials coded as references for the partial alignment of each scan. Known fringe patterns were projected onto the surface of the object and recorded by two cameras, based on the stereo camera principle. As the beam paths of both cameras and the projector were calibrated in advance (extrinsic calibration), 3D surface points from three different ray intersections could be calculated. The number of scans depended on the part surface complexity and required detail. Every partial scan was transformed to a common coordinate system employing as a reference the points that were measured before with the photogrammetric method. Once the part was totally scanned, the point cloud was transformed to a continuous mesh by means of Delaunay triangulation approaches [53,54,55,56].

## 3. Results

The following content presents the results of the comparison of the studied dense matching approaches against photogrammetrically referenced structured light 3D scanner for both lab and shop-floor lighting conditions. Moreover, for each different case study, the photogrammetric orientation problem was solved considering artificial coded targets or natural key features employing the same acquired image dataset.

One of the main differences between using the artificial coded targets or natural key features is the processing time and the automation level for photogrammetric model solving. Whereas the artificial coded target approach is totally automatic for camera network orientation estimation and scaling, the feature based targetless orientation approach requires a specific feature identification image parameter adjustment as well as manual 3D point cloud scaling. The processing time takes twice as long as using coded targets and increases exponentially with the number of images, feature point number and camera resolution. Coded target employment is a method to optimize this step, from the point of view of robustness and computing cost. The highest processing time is for dense matching reconstruction which takes several hours to obtain a full resolution 3D point cloud. The obtained accuracy is shown and discussed below for each case study.

To compare and assess the accuracy of the novel methodology, free 3D inspection software was used (GOM Inspect©, Braunschweig, Germany). The employed method for any comparison was the following one:

1. Import both meshes: the reference data measured by GOM© systems and the one achieved by dense photogrammetry.
2. Perform raw alignment between meshes with six nearly common points on the surface.
3. Perform best-fit alignment for accurate registration considering the reference data as nominal mesh.
4. Compare the 3D color map between aligned meshes and analyze deviations among measured points.
5. Perform statistical evaluation of achieved deviations with histograms (2σ).

In the case of camera network estimation by means of FBM, as this method offers rather high number of 3D points within the photogrammetric stage, the surface comparison is presented both for sparse point cloud and dense point cloud case studies. Although other alignment methods (geometric, datum-based, and 3-2-1) are available, best-fit approach has been chosen as it estimates the minimum average oversize that ensures that material exists in those areas of the piece to be machined. Moreover, it has been considered as a more statistical alignment method for the comparison as it gives the same weight to all surface points instead of fitting some geometric elements and taking them as a reference to define the coordinate system.

### 3.1. Lab Tests for Medium Size Parts

#### 3.1.1. Orientation Based on Artificial Targets

**Figure 12:**
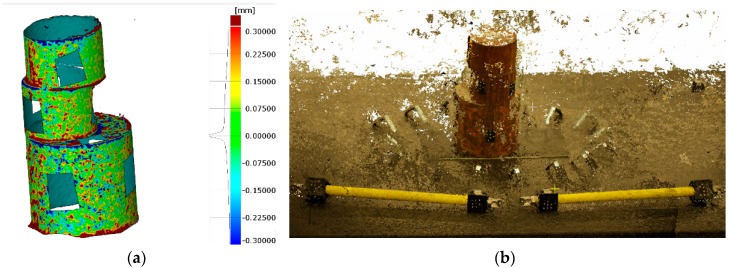
3D representation of measured part in lab environment with coded target orientation approach: (**a**) surface point comparison against reference mesh; and (**b**) dense point cloud with color and texture information (full resolution).

The first test was performed in lab conditions with stable lighting and environmental conditions. The part used for the experiment was an assembly of three rusted cylinders of Ø 50 mm × 500 mm in height with stochastic surface texture, as shown in Figure 12. After reference data acquisition (32 images), the overall dense photogrammetry was applied following the measuring procedure described in Section 2. The acquired images were initially used to solve the photogrammetry by means of coded-targets and then the dense point cloud reconstruction was established with high resolution.

The relative accuracy of the photogrammetry was 1/80,000 mm (RMS) and the camera self-calibration was accurately established. Regarding the surface data comparison, the deviations fell into ±0.2 mm (2σ), as shown in Figure 12a and its histogram. The obtained dense point cloud contained 10.5 million points (see Figure 12b), and coded targets were placed on lacking areas for determination of orientation. The higher deviation was found in edge points as the triangulation and the definition of the averaged points for dense matching is more challenging in these areas.

#### 3.1.2. Targetless Orientation Based on Feature Based Matching

The second test for lab conditions was performed on feature-based matching for camera network orientation establishment. The same pictures as in Section 3.1.1 were used but coded-targets were not employed to define the absolute orientation of the images. Once the photogrammetric part was solved, the data were scaled (sparse point cloud) and afterwards dense matching was done to obtain dense point cloud.

**Figure 13:**
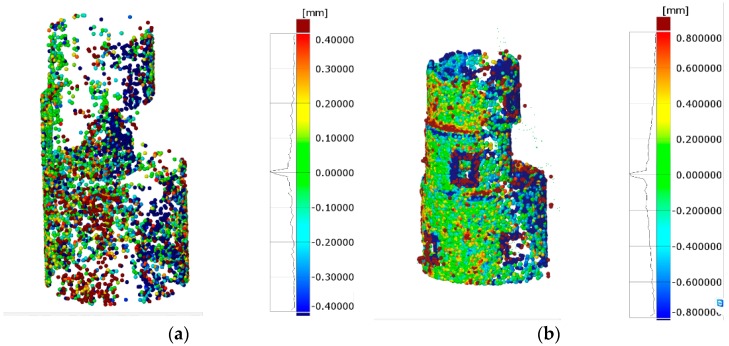
3D representation of measured part in lab environment with targetless orientation approach: (**a**) surface point comparison against reference mesh with sparse point cloud; and (**b**) surface point comparison against reference mesh with dense point cloud (quarter resolution).

The relative accuracy of the photogrammetry was 1/27,000 mm (RMS). Camera calibration was considered the same as obtained in the previous analysis due to higher accuracy assurance and convergence difficulties with self-calibration attempts. Regarding the surface data comparison, the deviations fell into ±0.4 mm (2σ), as shown in Figure 13a (sparse point cloud), and ±0.8 mm (2σ), as shown in Figure 13b (dense point cloud). The obtained dense point cloud contained 0.2 million points (see Figure 13b) with empty gaps were coded targets were placed. Again, the higher deviations were found in edge points and a difference of 200% was obtained comparing dense point cloud and sparse one.

### 3.2. Workshop Tests for Large Parts

#### 3.2.1. Orientation Based on Artificial Targets

**Figure 14:**
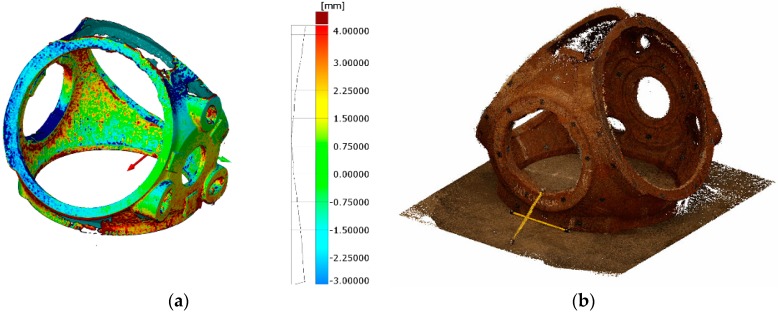
3D representation of measured part in shop-floor environment: (**a**) surface point comparison against reference mesh; and (**b**) dense point cloud with color and texture information (full resolution).

The preliminary test executed in workshop conditions did not assure stable lighting or proper accessibility to every part surface. The part used for the experiment was a casting eolic hub of 2500 mm × 2500 mm × 1500 mm with stochastic surface texture, as shown in Figure 14. After reference data acquisition (150 images), the dense photogrammetric was applied following the measuring procedure described in Section 5. The acquired images were initially used to solve the photogrammetry with coded-targets as in lab tests and then the dense point cloud reconstruction was determined.

The relative accuracy of the photogrammetry was 1/157,000 mm (RMS) and the camera self-calibration was accurately established. Regarding the surface data comparison, the deviations fell into ±3 mm (2σ), as shown in Figure 14a and its histogram. The obtained point cloud contained 2 million point with 0.2 mm resolution (see Figure 14b). The highest deviations were found in edge points, as occurred for lab results. The overall surface of the part was not scanned but most of the surfaces were measured, as shown in Figure 14b. To complete the scanning of every surface of the part, another part location is necessary, enabling the non-accessible surfaces to be measured. This requirement is usual for traditional measuring procedures but in this test a unique part location was applied and considered as enough for comparison.

#### 3.2.2. Targetless Orientation Based on Feature Based Matching

The second test for workshop conditions was performed on feature-based matching for camera network orientation establishment. The same pictures as in Section 3.2.1 were used but coded-targets were not employed to define the absolute orientation of the images. Once the photogrammetric part was solved, the data were scaled (sparse point cloud) and afterwards dense matching was carried out to obtain dense point cloud.

**Figure 15:**
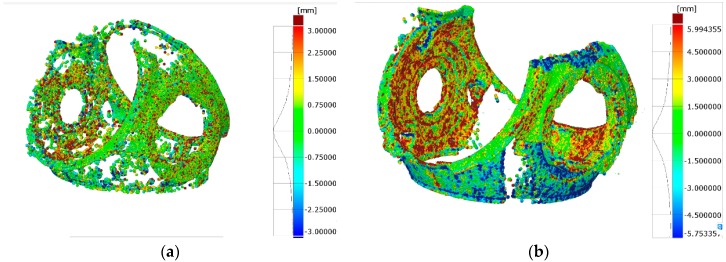
3D representation of measured part in shop-floor environment with targetless orientation approach: (**a**) surface point comparison against reference mesh with sparse point cloud; and (**b**) surface point comparison against reference mesh with dense point cloud (quarter resolution).

The relative accuracy of the photogrammetry was 1/38,500 mm (RMS). Camera calibration was considered the one obtained in the previous analysis due to higher accuracy assurance and convergence difficulties with self-calibration attempts. Regarding the surface data comparison, the deviations fell into ±3 mm (2σ), as shown in Figure 15a (sparse point cloud), and ±6 mm (2σ), as shown in Figure 15b (dense point cloud). The obtained dense point cloud contained 0.5 million points (see Figure 15b) with lacking areas at the upper part of the measurand. Again, the higher deviations were found in edge points and a difference of 200% was obtained comparing dense point cloud and sparse one.

## 4. Discussion

The trend in casting part dimensional inspection is to use smarter systems and procedures to speed up the measuring process assuring the acceptance criteria of the manufactured parts. Traditionally, measuring devices such as laser trackers or 3D optical scanners are used for FAI inspection or serial quality control. Time of flight based 3D laser scanners are also a recent alternative for 3D information acquisition of casting parts with uncertainties of about ±0.5 mm/m. Nevertheless, these devices do not allow automatic measurements because the measuring system has to be located and adjusted manually in different positions around the part. Partial measurements are placed in a unique coordinate system with the location of common artificial targets around the part.

Apart from this, other objectives such as improvement of the 3D point cloud processing step, automation of the measuring process, reduction of acquisition cost of the measuring devices, and robustness of the measuring process are also desired. This research was focused on studying the capabilities and scope of dense matching techniques and their suitability to be applied to casting part verification, where the manufacturing tolerances are not as tight as with machined parts. A comparison of dense matching approaches with traditional verification approaches is shown in Table 1 as a summary and overall overview of measuring alternatives’ pros and cons. The relative accuracy is compared taking into account only lab conditions and current standards as specifications of currently employed systems are defined in this environment.

The main advantages of studied methods as follows: fast acquisition time, high accessibility to multiple surfaces, high resolution (one point per pixel), high level of automation, and scalability to parts dimensions. The disadvantages are as follows: lower precision than other measuring techniques, the results contain points with noise, data are missing in the obtained point cloud depending on employed resolution and the extent is unknown until overall processing is carried out, high processing time, and expert user is required to ensure an accurate result in non-stable lighting environments.

Another important aspect related to this novel method is the low cost of the whole system comprising both the hardware and the software as well as the flexibility of the system for different scenarios and measuring scales. Moreover, the automation is feasible with a fixed multi-camera network set-up or CNC positioning systems enabling the possibility to develop inspection cells for manufacturing quality control. Currently, one important limitation to completely automatize the solution, is the offline processing step and high computation time for PCs. However, this drawback is day by day improving as the technology is constantly developing and smarter algorithms are being developed. Thus, in few years this limitation will be overcome and even real-time dense matching will be possible. Other aspects to be improved in further developments are the robustness of the technique from the point of view of rejection of outliers and data filtering to extract accurate results as well as development of standards and guidelines based on this technology.

Currently, advanced users are required to apply the measuring process described in this paper, but controlling lighting conditions can reduce this requirement and make more useful this procedure to all photogrammetric users. This way, this procedure could be offered as an alternative service for medium and large casting parts instead of applying other 3D approaches.

In relation to accuracy, dense matching techniques seem to be a suitable measuring procedure for casting part verification as they enable obtaining dense point cloud with accurate enough accuracy (lower than manufacturing tolerance) for part shape and dimension verification. The estimated relative accuracy (RMS) of the measured 3D point coordinates is determined as the ratio of the estimated point standard error over the effective maximum diameter of the object point array along with the corresponding proportional accuracy. Coded-target based approach is the most accurate approach. The grade of tolerances for casting is mentioned in ISO 8062-3:2007 Standard. This part of ISO 8062 defines a system of tolerance grades and machining allowance grades for cast metals and their alloys. It applies to both general dimensional and general geometrical tolerances, although this study was focused mainly on general dimensional verification. As a reference, for a common tolerance grade 12, tolerances of ±10 mm and ±17 mm are required for the dimensions of the parts analyzed in this study. Thus, obtained accuracies are fit to purpose considering these manufacturing tolerances.

A more detailed comparison of analyzed dense matching case studies is presented in Table 2, where obtained accuracy as well as required time for different measuring steps is shown as a summary of the results obtained in this research.

The relative accuracy (proportional RMS) for automated orientation based on coded-targets is 3–4 times higher compared with feature based matching approaches, for both lab and shop-floor case studies, whereas the deviations against traceable measuring methods are approximately twice lower. This difference is mainly due to the referencing step during the photogrammetric problem which is directly influenced by the accuracy of identification of key points for referencing. Whereas the automated orientation method employs the centroiding tool to estimate the center of the high-contrast elliptical targets with an accuracy of 0.03–0.1 pixels, targetless orientation approach uses less accurate (0.1–0.5 pixel) point identification methods, which leads to lower reconstructed point cloud accuracy and point cloud density. The final accuracy of 3D dense point cloud is a direct function of the referencing accuracy, so the more precise the point marking and referencing approach, the better the triangulation and the higher the point quantity of the reconstructed point cloud.

Higher image number and perspectives could also improve the triangulation of each reconstructed point and therefore the relative accuracy for both cases, but it will complicate the data processing phase, thus a compromise between accurate photogrammetry and suitable dense matching needs to be found. The number of reconstructed points totally depends on this balance as a more demanding point triangulation limit gives a lower density point cloud and vice versa. In this research, a minimum intersection of three viewpoints was required to estimate a 3D point. As the camera network orientation for targetless case is less accurate than the one with coded targets, the same parameterization for the point triangulation step gives lower dense point cloud and some missing areas.

The measuring time depends on the preparation of the scene and camera set-up as well as in the size of the part to be measured. However, comparing to other measuring methods, it is fast as only images of the part need to be taken from different perspectives and heights. One of the drawbacks of dense matching techniques is the data processing step once the images have been taken. Whereas photogrammetric problem estimation is affordable from the point of view of time compared to industrial solutions, dense matching step takes much time and PC memory, which makes it more difficult to process the same imagery with different image processing parameterization.

## 5. Conclusions

Edge-cutting dimensional verification technologies ensure a reliable dimensional assessment of casting parts to check if they fulfill the required conformity assessment. Low enough measuring uncertainties guarantee a proper part inspection, machining process and therefore an overall manufacturing process efficiency.

3D PCMMs are being used more and more nowadays for inspection tasks with advanced probes for data acquisition improvement, but they still require to be moved around the part which takes a long time for data acquisition. Besides, depending on part shape and dimensions, these techniques are hardly applicable to quality control requirements. Dense matching techniques based on imagery data seem to be an affordable alternative to these measuring techniques as they enable faster data acquisition schemas with rich data information. The processing of these data permits reconstructing dense point cloud with high relative accuracy. Therefore, volumetric surface data are estimated which enables surface data comparison against nominal CAD data.

However, this study should be complemented with other accuracy evaluation methods and applications where dense matching techniques are used for verification of textured industrial parts. Currently, few studies are contained in the literature from the metrological point of view that tackle measuring procedure uncertainty or traceability of these measuring techniques. For example, another procedure to establish the measuring accuracy could be to manufacture a calibrated part and use it as a known reference or to characterize each influence error to estimate the measuring uncertainty applying the “error propagation law” as it is carried out for calibration and adjustment of other metrological complex devices. The efficiency of data processing also needs improvement by utilizing parallel processing and hierarchical optimization techniques to quicken result procurement.

## Figures and Tables

**Table 1:** Summary and comparison of measuring techniques for casting part verification.

Measuring Device (PCMM)	Relative Accuracy (µm/m)	Surface Comparison	Accessibility	Scalability	Environmental Conditions	Ease of Use	Degree of Automation
Laser-tracker (ASME B89.4.19-2006)	±15 + 6 (MPE)	Low	Low	Low	Air flow stability required	High	Low
3D scanner (VDI 2634 BLATT 3)	±30	High	Low	Low	Laser stability	High	Low
Dense photogrammetry (Orientation with coded-targets)	1/80,000 (RMS)	High	High	High	Lighting stability required	Low	High
Dense photogrammetry (Targetless orientation)	1/27,000 (RMS)	High	High	High	Lighting stability required	Low	Medium to low

**Table 2:** Summary and comparison of dense matching techniques for lab and shop-floor environments.

Test Type	Point Number (million)	Relative Accuracy (mm/m)	Measuring Time (min)	Processing Time (min)		Manufacturing Tolerance Grade 12 (mm)	Deviations (2σ)	
				a	b		Sparse (mm)	Dense (mm)
Lab test 1	10.5	1/80,000	5	5	120 to 180	±10	-	±0.2
Lab test 2	0.2	1/27,000		12			±0.4	±0.8
Shop-floor test 1	2	1/157,000	15	10	>300	±17	-	±3
Shop-floor test 1	0.5	1/38,500		20			±3	±6

a: Orientation stage; b: Dense reconstruction.
